# Supplementary material for: The Progress of the New South Wales Aboriginal Oral Health Plan 2014–2020: A Scoping Review
Source: Healthcare (Basel). 2022 Mar 30;10(4):650. doi: 10.3390/healthcare10040650 (PMC9031810; doi:10.3390/healthcare10040650)
Supplement: Supplementary file 1 [file healthcare-10-00650-s001.zip › healthcare-1612637 supplementary.pdf]

Medline search strategy

Ovid MEDLINE(R) ALL <1946 to February 14, 2020>			
**#**	**Search Statement**	**Results**	**Annotation**
1	Oceanic Ancestry Group/	9986	
2	aborigin*.mp.	9763	
3	indigenous.mp.	32827	
4	torres strait*.mp.	1712	
5	first nation*.mp.	4507	
6	ATSI.mp.	59	
7	1 or 2 or 3 or 4 or 5 or 6	47391	
8	New South Wales/	12616	
9	new south wales.mp.	16445	
10	8 or 9	16445	
11	Oral Health/	16067	
12	(oral health adj2 (plan* or promotion* or program* or intervention* or service*)).mp.	2948	
13	(dental health adj2 (plan* or promotion* or program* or intervention* or service*)).mp.	5071	
14	Dental Health Services/	4062	
15	11 or 12 or 13 or 14	21579	
16	7 and 10 and 15	24	
17	limit 16 to last 5 years	13	

Execute Searches in Ovid
